# Beyond Ablation: A Review of Immune Responses Across Focused Ultrasound Modalities

**DOI:** 10.3390/cancers18152460

**Published:** 2026-07-31

**Authors:** Carley M. Elliott, Tamalika Paul, Michaela Hall, Sofia Killar, Eli Vlaisavljevich, Irving C. Allen

**Affiliations:** 1 Graduate Program in Translational Biology, Medicine, and Health, Virginia Tech, Roanoke, VA 24016, USA; carleye6@vt.edu (C.M.E.); michaelafh@vt.edu (M.H.); smkillar@vt.edu (S.K.); 2 Department of Biomedical Sciences and Pathobiology, Virginia-Maryland College of Veterinary Medicine, Blacksburg, VA 24061, USA; tamalika@vt.edu; 3 Department of Biomedical Engineering and Mechanics, Virginia Polytechnic Institute and State University, Blacksburg, VA 24061, USA; eliv@vt.edu; 4 College of Science, Virginia Tech, Blacksburg, VA 24061, USA

**Keywords:** focused ultrasound (FUS), high-intensity focused ultrasound (HIFU), histotripsy, boiling histotripsy, low-intensity focused ultrasound (LIFU), tumor microenvironment (TME), immunomodulation, cavitation, immunogenic cell death (ICD), anti-tumor immunity

## Abstract

Focused ultrasound (FUS) comprises a diverse group of non-invasive, non-ionizing acoustic technologies that have evolved from tools for localized tissue destruction into platforms capable of influencing complex biological processes. In oncology, growing evidence suggests that the effects of focused ultrasound extend beyond direct tumor treatment to include modulation of the tumor microenvironment and anti-tumor immunity. This review examines the major FUS modalities currently under investigation for cancer therapy, including high-intensity focused ultrasound (HIFU), intrinsic threshold histotripsy, boiling histotripsy, shock-scattering histotripsy, and low-intensity focused ultrasound (LIFU), with emphasis on the distinct physical mechanisms that underlie their biological effects. Although these modalities differ in how they interact with tissue, they share the capacity to alter tumor biology through changes in antigen availability, inflammatory signaling, and immune cell activity. These responses have been associated with enhanced immune recognition of tumors, remodeling of immunosuppressive microenvironments, and improved therapeutic responsiveness in preclinical and emerging clinical studies. As interest in focused ultrasound continues to expand, understanding the relationship between modality-specific bioeffects and downstream immune outcomes has become increasingly important. Collectively, the literature highlights focused ultrasound as a versatile therapeutic platform capable of linking precise local intervention with broader biological and immunological consequences, supporting its continued development as both a tumor-directed and immune-modulating strategy in cancer therapy.

## Simple Summary

Focused ultrasound is an emerging non-invasive technology that can treat tumors using precisely targeted ultrasound waves. Different forms of focused ultrasound can destroy tumor tissue through heat, mechanical forces, or temporary changes in tissue function, offering alternatives to conventional cancer treatments. Growing evidence suggests that these approaches may do more than remove tumors locally; they may also promote anti-tumor immune responses by enhancing immune recognition of cancer cells. This review examines the major focused ultrasound technologies used in oncology, highlighting how their distinct mechanisms influence tumor biology and anti-cancer immunity. By comparing these approaches, the review aims to provide a clearer understanding of their potential to improve cancer treatment outcomes and support the development of combination therapies, including immunotherapy. These insights may help guide future research and accelerate the clinical translation of focused ultrasound technologies for cancer care.

## 1. Introduction

Focused ultrasound (FUS) encompasses a diverse group of non-invasive, non-ionizing acoustic technologies capable of producing highly distinct biological effects through modulation of ultrasound parameters and tissue interactions. Rather than representing a single therapeutic approach, FUS includes multiple modalities that span thermal ablation, mechanical tissue fractionation, cavitation-based destruction, neuromodulation, and transient vascular or blood–brain barrier (BBB) disruption. The modalities discussed in this review include high-intensity focused ultrasound (HIFU), intrinsic threshold histotripsy, boiling histotripsy, shock-scattering histotripsy, and low-intensity focused ultrasound (LIFU), each of which utilizes unique acoustic mechanisms to achieve different therapeutic outcomes.

**Figure 1:**
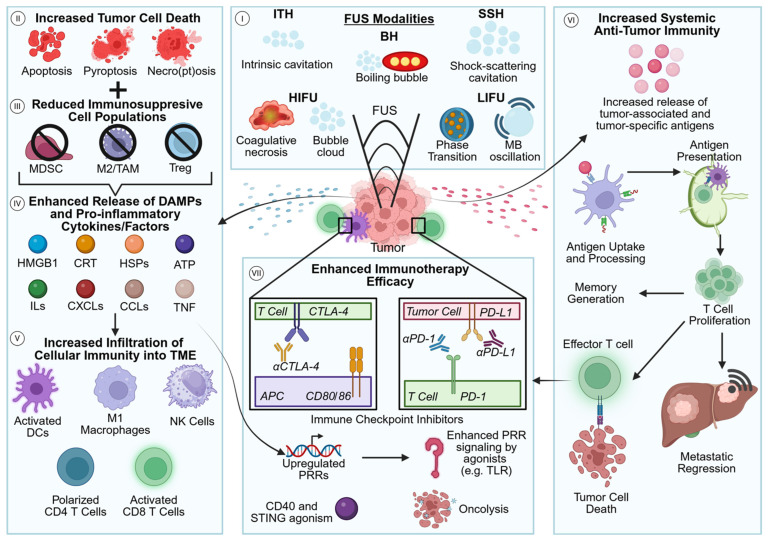
Conceptual model of potential immunologic mechanisms underlying FUS-mediated antitumor immunity. Distinct FUS modalities may induce thermal and/or mechanical tumor disruption, potentially resulting in tumor cell death, the release of DAMPs, cytokines, and tumor antigens, and subsequent remodeling of the tumor microenvironment (TME). These changes are proposed to promote innate and adaptive immune activation, including antigen presentation, T-cell priming, and systemic antitumor immune responses. In turn, FUS-mediated immune stimulation may enhance the efficacy of immunotherapies, including immune checkpoint blockade and other immune agonists. Not all pathways depicted have been experimentally demonstrated for every FUS modality and should be interpreted as a conceptual synthesis of the current literature.

Initially developed primarily for localized tissue ablation, focused ultrasound modalities have increasingly gained attention for their ability to modulate the tumor microenvironment (TME) and influence anti-tumor immune responses. Thermal approaches such as HIFU induce coagulative necrosis through localized heat deposition, whereas histotripsy-based modalities mechanically disrupt tissue through cavitation bubble dynamics and shockwave interactions [1,2,3,4,5,6,7,8,9,10,11,12,13,14,15]. In contrast, non-ablative approaches such as LIFU can transiently alter vascular permeability, BBB integrity, and cellular signaling without extensive tissue destruction [16,17,18]. These differing mechanisms result in distinct patterns of tissue injury, antigen release, inflammatory signaling, and immune activation. The hypothesized relationships between focused ultrasound-mediated tissue disruption, immune activation, and therapeutic synergy with immunotherapy are summarized in Figure 1.

Across multiple preclinical and clinical studies, focused ultrasound modalities have been associated with tumor antigen release, induction of damage-associated molecular pattern (DAMP) signaling, dendritic cell activation, and recruitment of fundamental innate and adaptive immune cell populations. As a result, these technologies are increasingly being investigated for their ability to influence tumor biology beyond local tumor control, particularly through modulation of the TME and anti-tumor immune responses.

This review summarizes the major focused ultrasound modalities currently under investigation for oncologic applications, emphasizing their distinct biophysical mechanisms, effects on the local and systemic immune environments, and expanding roles beyond traditional tumor ablation. A comparative summary of the major immune responses and translational applications associated with each modality is provided in Table 1. By highlighting both shared and modality-specific immune responses, this review aims to identify emerging principles that may guide the rational development of focused ultrasound-based cancer therapies and combination treatment strategies.

## 2. High-Intensity Focused Ultrasound (HIFU)

Over the past several decades, high-intensity focused ultrasound (HIFU) has emerged as a widely investigated non-invasive, non-ionizing ablation modality for the treatment of solid malignancies [1]. Rather than relying on a single mechanism of action, HIFU induces tissue injury through a continuum of coupled mechanical and thermal effects that are determined by exposure parameters and tissue context. Acoustic energy is delivered through overlying tissues without damage and converges at a focal point, where it can be applied in continuous modes to drive thermal deposition or pulsed modes that favor acoustic cavitation (Table 1). Cavitation involves the formation and rapid oscillation of gas-filled microbubbles under high-amplitude pressure fluctuations, generating localized mechanical stresses that disrupt cellular and extracellular structures [2]. Taken together, these mechanisms establish HIFU as a modality capable of producing controlled, spatially confined tissue injury through both thermal and non-thermal pathways.

These biophysical effects converge to generate predictable zones of tumor destruction characterized by distinct thermal and structural outcomes. Thermal energy deposition within the focal region can elevate tissue temperatures to ≥56 °C, resulting in coagulative necrosis through protein denaturation, cellular destruction, and structural stiffening when sustained over clinically relevant durations [3,4,5,6]. This process yields a sharply demarcated boundary between ablated and viable tissue, reflecting the high spatial precision of energy delivery. The integration of thermal and mechanical injury mechanisms positions HIFU as a precise ablative platform with the capacity to reshape the tumor microenvironment and influence downstream immunomodulatory pathways.

As the oncological applications of HIFU continue to expand, increasing evidence demonstrates that beyond tumor debulking, HIFU can induce substantial remodeling within the tumor microenvironment. Certain malignancies present unique therapeutic challenges due to highly immunosuppressive TMEs, dense stromal architecture, and heterogeneous cellular composition, all of which can limit therapeutic penetration, reduce immune cell infiltration, and contribute to treatment resistance and tumor recurrence. However, preclinical studies have demonstrated that HIFU can promote restructuring of the local immune landscape following treatment, including shifts from an immunologically “cold” anti-inflammatory environment toward an immunologically “hot” pro-inflammatory environment that may support localized immune activation (Table 1) [19,20,21,22,23]. The conceptual pathways linking FUS-induced tissue disruption to local and systemic immune responses are summarized in Figure 1.

Among the most consistently observed changes following HIFU treatment is the increased infiltration of CD4^+^ and CD8^+^ T cells within treated tumors, particularly in preclinical neuroblastoma and pancreatic cancer models [19,20]. This influx of both “helper” and “cytotoxic” T cells reflects enhanced CD4^+^ T-cell-mediated coordination of antigen presentation, cytokine production, and immune cell recruitment alongside increased CD8^+^ T-cell recognition and targeting of tumor-associated antigens (TAAs) within the local TME following HIFU treatment. This enhanced lymphocyte infiltration may also reflect HIFU-induced upregulation of chemokines and adhesion molecules that facilitate T-cell trafficking into the treated tumor microenvironment, further supporting the establishment of localized antitumor immunity [24,25,26]. Concurrently, increased macrophage infiltration has also been documented across multiple preclinical models, indicating active remodeling of the innate immune compartment, enhanced phagocytic clearance of cellular debris, and the potential for altered inflammatory signaling within the post-ablation TME [20,21].

In addition to lymphocyte and macrophage recruitment, dendritic cell (DC) accumulation has been observed in HIFU-treated tumors, particularly along the peripheral regions of thermal lesions [22]. These findings expanded upon earlier work demonstrating HIFU-associated upregulation of heat shock proteins (HSPs) and their potential role in promoting DC maturation within treated tissues [22,23]. Together, these observations suggest that HIFU may facilitate the initiation of adaptive immune responses by enhancing antigen capture, processing, and presentation within the treated TME (Table 1). Further investigation into the immunostimulatory effects of HIFU in a 4T1 murine breast cancer model demonstrated increased markers of immunogenic cell death (ICD), including the DAMPs calreticulin (CRT), ATP, and high mobility group box 1 protein (HMGB1) [27]. The increased exposure and release of these DAMPs prompted subsequent studies investigating their role in DC activation and maturation. Analysis of major histocompatibility complex class II (MHC II) expression and the co-stimulatory molecule CD86, both established markers of DC maturation, recapitulated findings from earlier studies demonstrating that HIFU treatment significantly increased markers associated with mature DC phenotypes [22,23,27].

While these findings demonstrate substantial remodeling of the local TME following HIFU treatment, many of the earliest post-ablation events are governed by activation of innate immune sensing pathways that translate treatment-induced disruption into molecular inflammatory signals. This response is initiated by rapid detection of cellular stress and structural damage through pattern recognition receptors (PRRs), which recognize DAMPs released from ablated tumor cells (Table 1). Engagement of these sensors activates downstream inflammatory signaling cascades that establish the foundational innate immune response following HIFU treatment.

Consistent with this, HIFU ablation has been shown to induce broad upregulation of innate immune sensing machinery, including Toll-like receptors (TLRs), NOD-like receptors (NLRs), cytosolic DNA sensors, and RIG-I-like receptors (RLRs) [28,29,30,31]. In a syngeneic HER2-driven mammary adenocarcinoma model derived from the neu exon deletion line (NDL), HIFU was further associated with activation of multiple sensing axes, including NLRP3 and AIM2 inflammasome components, cytosolic nucleic acid sensors, and stress-associated mediators such as OAS2 and RhoA [28]. This coordinated induction reflects the conversion of mechanical and/or thermal tumor injury into a broad pattern-recognition–driven inflammatory signaling network, enabling rapid detection of DAMP-associated cues and initiation of downstream innate signaling programs [28]. 

Downstream of these sensing events, inflammasome-associated signaling is prominently engaged, as evidenced by increased interleukin-1β (IL-1β) expression following HIFU ablation in an NDL murine model. This upregulation is linked to caspase-1–dependent processing and is associated with inflammatory programming consistent with Th1-skewing immune polarization. In parallel, interleukin-1α (IL-1α) functions as a DAMP itself, capable of engaging TLR signaling to further amplify inflammatory cascade activation [28,32]. These pathways highlight the integration of inflammasome activity with TLR-driven signaling as a central feature of the innate response to HIFU ablation.

Beyond canonical PRR pathways, HIFU also induces tumor cell–intrinsic transcriptional reprogramming. In a 4T1 breast cancer model, treatment has been shown to enrich immune- and stress-associated signaling pathways, including IL-17, MAPK, PPAR, and TNF signaling pathways [33]. These changes reflect activation of conserved inflammatory and stress-response programs within tumor cells themselves, further reinforcing the innate signaling environment generated in response to HIFU treatment. Following the initial innate immune sensing and inflammatory signaling induced by HIFU, these early events converge to facilitate the initiation of adaptive immune responses through antigen processing and presentation within lymphoid tissues. This transition is characterized by the mobilization of tumor-derived antigens and their engagement with antigen-presenting cells (APCs), ultimately linking localized injury to the activation and expansion of antigen-specific lymphocyte populations.

HIFU treatment has been documented to generate tumor-associated antigens across a variety of cancer subtypes, supporting its role in initiating antigen-driven immune priming. In a clinical study of prostate cancer patients treated with HIFU, ex vivo immune assays demonstrated the induction of tumor antigen-specific T-cell responses following treatment. Peripheral blood mononuclear cells isolated after HIFU exposure showed increased reactivity against prostate-associated antigens, including PSA, prostate-specific antigen, and PSMA, prostate-specific membrane antigen, when co-cultured with autologous dendritic cells exposed to these peptides [34]. Notably, both CD4^+^ and CD8^+^ T-cell populations exhibited enhanced antigen-specific activation post-treatment, indicating that HIFU-mediated tumor ablation promotes the generation and/or increased availability of TAAs that can be processed and presented to the adaptive immune system (Table 1) [34].

As responses to HIFU treatment extend systemically, adaptive immune changes have also been observed within key secondary lymphoid sites, including tumor-draining lymph nodes (TDLNs). In a clinical breast cancer study, TDLNs demonstrated increased populations of T lymphocytes and subsets, B lymphocytes, and NK cells [35]. Specifically, CD3^+^, CD4^+^, and CD57^+^ cells were more frequent in HIFU-treated groups, alongside cytotoxic T cells expressing FasL, GzB, and Pf, which were also detected in metastatic-positive axillary lymph nodes [35]. Collectively, these findings indicate that HIFU treatment promotes systemic, antigen-driven adaptive immune activation characterized by T-cell expansion, cytotoxic differentiation, and lymphoid engagement (Table 1). While these studies collectively support the immunomodulatory potential of HIFU, interpretation of the current literature is complicated by substantial variability in tumor models, ultrasound exposure parameters, and reporting practices. Differences in acoustic frequency, pressure, duty cycle, treatment duration, tissue characteristics, and inconsistent parameter reporting make direct comparisons between studies difficult and limit identification of the treatment conditions responsible for specific immune outcomes [36]. These limitations underscore the need for standardized treatment protocols and additional clinical validation to facilitate broader translation of HIFU-based therapies into routine oncologic practice.

Despite these remaining challenges, HIFU has demonstrated broad clinical utility across oncologic and neurologic disease, reflecting its role as a versatile non-invasive therapeutic platform. In oncology, early clinical studies in the late 1990s established the feasibility of safely ablating liver lesions with minimal toxicity [37]. Subsequent trials in unresectable hepatic tumors further supported its effectiveness for local tumor control, particularly with MRI and advanced ultrasound guidance [38,39,40]. In pancreatic cancer, HIFU has been associated with survival benefits across multiple studies, including reduced cancer-related pain and improved localized tumor control [41,42,43]. Similar clinical benefit has been reported in renal and breast tumors. In renal cancer, HIFU has demonstrated pain relief, tumor reduction, and successful ablation in both primary and metastatic disease, with reported median survival ranging from 14.1 to 18.5 months [44,45,46,47,48]. In breast tumors, HIFU has achieved high rates of coagulative necrosis and tumor reduction with minimal severe complications, with MRI-guided approaches reporting necrosis rates nearing 97% and low recurrence at 12 months [49,50,51]. Breast fibroadenomas have also shown sustained volume reduction with mild transient skin-related adverse effects [52,53]. Beyond oncology, MRI-guided HIFU has shown clinical efficacy in neurologic disorders. In essential tremor, HIFU significantly improved tremor severity, disability, and quality of life with durable benefit up to 12 months [54,55,56]. In Parkinson’s disease, HIFU pallidotomy and thalamotomy improved motor symptoms and quality of life, with mostly transient adverse effects [57,58,59,60] and has also demonstrated benefit in chronic therapy-resistant neuropathic pain [61]. HIFU is also increasingly being explored for BBB disruption to enhance drug delivery, with additional investigational applications in Alzheimer’s disease, epilepsy, obsessive–compulsive disorder, and neuromodulation [62,63,64,65,66,67]. Preclinical studies have demonstrated that HIFU can also function as an immunomodulatory therapy (Table 1). When combined with CTLA-4 and PD-L1 checkpoint blockade, HIFU significantly improved survival, enhanced infiltration of intratumoral CD4^+^ and CD8^+^ T cells and dendritic cells, promoted activation of NK cells and pro-inflammatory cytokine production, and reduced immunosuppressive mediators including IL-10, VEGF-A, and regulatory T cells [19]. Importantly, combination therapy has also been associated with robust abscopal responses and the generation of transferable effector-memory T-cell populations capable of delaying subsequent tumor engraftment, highlighting the potential of HIFU to synergize with immunotherapy and induce durable systemic antitumor immunity [19]. These findings support HIFU as an expanding translational platform across oncology and neurology.

## 3. Intrinsic Threshold Histotripsy

Histotripsy is a non-invasive, ultrasound-based ablative technology that mechanically destroys tissue using focused acoustic energy. The term itself derives from “histo,” meaning tissue, and “tripsy,” meaning to break apart. In contrast to thermal ablation techniques, including radiofrequency ablation and HIFU, histotripsy does not rely on heat-mediated cytotoxicity for tumor destruction. Instead, it utilizes short, high-pressure ultrasound pulses to induce acoustic cavitation within targeted tissue. This process is driven by the formation, oscillation, and collapse of cavitation bubbles generated under alternating cycles of positive and negative pressure. When the applied negative pressure exceeds a critical threshold, microscopic gas nuclei expand rapidly and subsequently collapse within microseconds, producing intense localized mechanical forces. These forces are sufficient to disrupt cellular membranes and extracellular structures, ultimately resulting in liquefaction and homogenization of the targeted tissue without thermal injury (Table 1) [7,8,9].

A defining feature of histotripsy is its precision and threshold-dependent activity. Tissue ablation occurs only when acoustic pressures exceed a defined cavitation threshold, enabling highly localized treatment zones with sharp demarcation between treated and untreated tissue. This spatial precision is particularly advantageous for tumors located adjacent to critical anatomical structures, including blood vessels, bile ducts, and nerves. Importantly, histotripsy has also been shown to preferentially disrupt soft tumor tissue while sparing more collagen-dense structures, potentially reducing treatment-associated complications. Additional advantages include its non-invasive and non-ionizing nature. Unlike radiation therapy, which exposes surrounding tissues to ionizing radiation and associated long-term toxicity risks, histotripsy relies solely on mechanical ultrasound energy. Furthermore, the absence of surgical incision reduces procedural morbidity, infection risk, and recovery time [7,8,9].

Histotripsy has also been shown to substantially remodel TME, primarily through mechanical disruption of tumor architecture [68]. Acoustic cavitation fragments cellular and extracellular structures, dismantling the structural framework that supports tumor growth and immune exclusion. This process facilitates exposure of tumor antigens and inflammatory mediators that are otherwise physically sequestered from immune recognition [68]. Across multiple tumor models, histotripsy has been associated with a shift from a “cold” anti-inflammatory phenotype to a more inflammatory or “hot” phenotype (Table 1). Notably, pancreatic tumors, which are typically characterized by poor immune infiltration and attenuated inflammatory signaling, also exhibited increased immune cell recruitment and enhanced inflammatory activity following treatment [68,69].

In parallel, histotripsy may modulate additional features of the TME that contribute to immune resistance. Hypoxia, a hallmark of aggressive and therapy-resistant tumors, suppresses effector immune function and promotes tumor survival pathways [70]. Emerging evidence suggests histotripsy may reduce hypoxic signaling, thereby improving immune cell functionality and therapeutic susceptibility. Similarly, histotripsy may diminish immunosuppressive cell populations that are commonly recruited by tumors to limit cytotoxic immune activity, thereby shifting the local balance toward anti-tumor immunity [70,71,72]. Beyond structural disruption, histotripsy generates an immunologically active milieu enriched in tumor antigens, cytokines, and inflammatory mediators [68,69,70,71,72,73,74]. These factors promote recruitment of APCs, including dendritic cells and macrophages, supporting downstream immune activation. These effects have motivated increasing interest in combining histotripsy with immunotherapeutic strategies. By enhancing antigen availability and inflammatory signaling, histotripsy may improve responsiveness to immune checkpoint blockade and help recondition immunologically resistant tumors. Overall, histotripsy not only achieves local tumor destruction but also reshapes the TME in ways that support immune activation while limiting tumor-promoting processes [68,69,70,71,72,73,74].

The innate immune system represents the first line of defense against tissue injury and pathogenic insult and plays a central role in the biological response to histotripsy. A key initiating mechanism is the release of DAMPs following mechanical tumor fragmentation. During treatment, tumor cells are physically disrupted, resulting in the liberation of intracellular molecules that are normally sequestered within intact cells, including HMGB1, ATP, HSPs, and nucleic acid fragments. Once released into the extracellular space, these DAMPs function as immunological danger signals indicative of tissue injury. DAMPs are rapidly detected by PRRs on innate immune cells, including macrophages, dendritic cells, and neutrophils. These receptor systems include TLRs, NLRs, and RLRs. Engagement of these pathways initiates downstream inflammatory signaling cascades that promote cytokine production, immune cell recruitment, and antigen presentation (Table 1) [68,71,75]. Macrophages and dendritic cells subsequently process tumor-derived antigens released during ablation, linking innate sensing to downstream adaptive immune activation. Importantly, because histotripsy is non-thermal, it has been hypothesized that native tumor antigenic structures are preserved following ablation. This contrasts with thermal ablation approaches that may denature proteins and reduce antigen availability for immune recognition [4].

Innate immune activation is further characterized by robust cytokine production, including tumor necrosis factor-alpha (TNF-α), interleukin-6 (IL-6), interleukin-1β (IL-1β), and interferon-gamma (IFN-γ) [72]. These mediators amplify inflammatory signaling, support immune cell recruitment, and coordinate communication between innate and adaptive immune responses (Table 1) [71]. In parallel, histotripsy may modulate vascular and stromal components within the tumor, increasing vascular permeability and facilitating immune cell access to tumor-derived antigens. While this inflammatory response is a key component of its therapeutic mechanism, the balance between beneficial immune activation and excessive inflammation remains an important area of investigation. Taken cumulatively, current evidence strongly supports innate immune activation as a central driver of histotripsy’s biological effects [71,76]. These findings align with the conceptual model of FUS-mediated immune activation illustrated in Figure 1.

Although innate immune activation regulates the immediate inflammatory response to histotripsy, adaptive immunity underlies the generation of antigen-specific and durable anti-tumor effects. A key emerging concept is that localized tumor ablation can initiate systemic immune activation. Adaptive responses are initiated when antigen-presenting cells process tumor-derived antigens released during treatment and present them within lymphoid tissues. This leads to the activation and expansion of tumor-specific T cells, particularly CD8^+^ cytotoxic T lymphocytes, which are central to tumor recognition and elimination (Table 1). Preclinical studies have demonstrated increased CD8^+^ T-cell infiltration following histotripsy, consistent with enhanced adaptive immune engagement [77]. Once activated, these effector T cells can circulate systemically and contribute to the control of residual or metastatic disease beyond the primary treatment site. Histotripsy has also been associated with the development of immunological memory, with long-lived memory T-cell populations capable of rapid reactivation upon tumor re-exposure, supporting durable immune surveillance and reduced recurrence risk [69]. These systemic adaptive effects are particularly relevant in metastatic disease, where localized interventions are insufficient for disease control. In addition, histotripsy may attenuate intratumoral immunosuppressive signaling pathways that limit T-cell activity, providing a mechanistic basis for its growing investigation in combination with immune checkpoint inhibitors such as anti-PD-1, anti-PD-L1, and anti-CTLA-4 therapies (Table 1). By enhancing antigen availability and inflammatory signaling, histotripsy may improve responsiveness to these immunotherapeutic strategies [9,78,79,80,81,82].

Histotripsy has rapidly transitioned from experimental development into a clinically relevant therapeutic modality with applications across multiple cancer types. One of the most established applications is in the treatment of liver tumors, including both primary hepatocellular carcinoma and metastatic lesions. Currently, thermal ablation techniques such as radiofrequency ablation (RFA) remain the standard locoregional treatment for many unresectable liver tumors. However, because RFA requires percutaneous probe placement and relies on thermal energy, its efficacy may be limited by the heat-sink effect near major blood vessels and the risk of thermal injury to adjacent critical structures [83]. In contrast, histotripsy mechanically fractionates tissue without thermal injury or needle insertion, offering a completely non-invasive alternative that may overcome some of these limitations while preserving the potential for immune activation. The HOPE4LIVER trial demonstrated high technical success rates and favorable safety outcomes, supporting histotripsy as a viable non-invasive treatment option for hepatic malignancies [82,84,85]. Follow-up imaging further confirmed gradual reduction in ablation zones, consistent with tissue resorption and healing processes [84]. Beyond liver cancer, histotripsy is also under investigation for pancreatic cancer, melanoma, kidney tumors, and prostate cancer. Histotripsy is currently being evaluated in pancreatic cancer patients through the ongoing GANNON trial (NCT06282809), which aims to establish the safety and feasibility of this technique. Its precision and non-invasive nature make it particularly promising for anatomically complex or surgically challenging tumor locations. One of the most compelling emerging concepts in histotripsy research is its potential to induce abscopal effects, whereby localized treatment leads to regression of distant, untreated tumors (Table 1). Although evidence in humans remains limited, a recent clinical case report described regression of untreated liver metastases following histotripsy of a single hepatic lesion, accompanied by sustained reductions in carcinoembryonic antigen levels. While causality cannot be established from a single case, these findings provide preliminary clinical support for the systemic immune activation and abscopal responses previously observed in preclinical models [86]. This phenomenon is thought to be immune-mediated and highlights histotripsy’s capacity to function as a systemic immunological trigger rather than solely a local ablative therapy. Additionally, histotripsy is being explored as a form of in situ immunotherapy, leveraging localized tumor destruction to stimulate systemic immune activation. This approach may reduce reliance on systemic drug administration and associated toxicities. Because histotripsy is non-invasive, it is also associated with reduced recovery time, lower complication rates, and potential for outpatient-based delivery, further enhancing its clinical utility [80,87,88]. Despite this rapid progress, intrinsic threshold histotripsy remains an evolving therapeutic platform. While preclinical studies have consistently demonstrated encouraging biological and immunological activity, continued refinement of treatment strategies will be important as the field advances toward broader clinical implementation. Because cavitation behavior and lesion formation are intrinsically dependent on acoustic exposure conditions, additional studies are needed to optimize treatment parameters and establish standardized protocols across tumor types [89]. Furthermore, larger prospective clinical studies will be essential to validate efficacy and define the role of intrinsic threshold histotripsy in routine oncologic care. 

## 4. Boiling Histotripsy

Boiling histotripsy is a focused ultrasound modality that employs high-intensity acoustic pulses to generate boiling vapor bubbles and localized shockwaves, producing mechanical tissue disruption [10,11]. In contrast to conventional HIFU approaches that rely on microsecond pulses, boiling histotripsy employs repetitive millisecond pulses that extend just beyond the time-to-boil threshold, heating tissue to approximately 100 °C and producing tissue emulsification through explosive boiling and shock wave interactions (Table 1) [11]. These shock waves exhibit high positive peak pressures (40–80 MPa) with comparatively lower negative pressures than cavitation-based histotripsy, enabling controlled boiling bubble formation while reducing transducer requirements [11]. Although shockwave heating contributes to bubble formation, boiling histotripsy is considered non-thermal in its biological effect, as tissue disruption is driven primarily by mechanical emulsification with minimal thermal injury, thereby reducing heat-sink limitations [11].

Boiling histotripsy has been validated across a range of ex vivo tissue models, including prostate, cardiac, and hepatic tissues, as well as solid tumor types such as prostate adenocarcinoma, breast carcinoma, and leiomyoma [11,90,91,92,93,94]. In vivo studies have further demonstrated feasibility using MR-guided and B-mode ultrasound imaging to monitor treatment parameters and assess ablation efficacy across multiple tissue types [95,96]. Histologic analyses consistently demonstrate liquefactive lesions confined to the treatment zone, with preservation of structural components such as vascular architecture, supporting the non-thermal and mechanically selective nature of boiling histotripsy. Owing to these properties, boiling histotripsy has been proposed as a strategy for preserving native tumor antigens and enabling immune stimulation, in addition to supporting the development of smaller, potentially handheld or endoscopic transducers for translational applications (Table 1) [91,97].

Beyond mechanical tissue liquefaction, boiling histotripsy induces substantial remodeling of the TME, shifting it from an immunologically “cold” to a “hot” inflammatory state (Table 1). This transition is driven in part by preservation of tumor antigens and their subsequent recognition by APCs, alongside activation of necrotic and inflammatory signaling pathways. Early after boiling histotripsy treatment, damaged cells release DAMPs, which are detected by PRRs and initiate downstream inflammatory signaling and cell death pathways (Table 1).

In renal cell carcinoma, boiling histotripsy treatment induces rapid increases in DAMPs, including calreticulin, HSPs, and HMGB-1, alongside activation of TNF signaling pathways, without concurrent activation of canonical apoptotic markers such as caspases, FAS, or FASLG [98]. This pattern is consistent with TNF-mediated necrotic signaling contributing to enhanced tumor cell death within the treated region [98]. DAMP-associated signaling also promotes pro-inflammatory cytokine release and recruitment of innate and adaptive immune populations, including macrophages, dendritic cells, and CD8^+^ T cells (Table 1). In melanoma models, boiling histotripsy enhances dendritic cell activation and antigen trafficking from the TME to TDLNs within 18 h post-treatment [99]. This facilitates CD8^+^ T-cell activation and supports CD4^+^ T-cell priming and expansion, promoting cytokine production and clonal proliferation [99,100]. In parallel, boiling histotripsy increases pro-inflammatory M1 macrophages while reducing immunosuppressive M2 macrophages (Table 1) [98,100]. In pancreatic cancer, boiling histotripsy also promotes a shift from N2 to N1 neutrophil phenotypes and reduces T regulatory cell populations, further reinforcing a pro-inflammatory TME [101]. These changes reflect coordinated remodeling of TNF-driven necrosis, cytokine signaling, and immune infiltration across multiple innate and adaptive immune compartments.

Innate immune activation following boiling histotripsy is characterized by robust inflammatory signaling, cytokine production, and induction of cell death programs. In renal cell carcinoma, boiling histotripsy treatment significantly increases pro-inflammatory cytokines, including IFN-γ, IL-1α, IL-1β, IL-18, and IL-8 compared with controls [98]. In orthotopic pancreatic cancer models, boiling histotripsy induces upregulation of type I interferon signaling and increased expression of innate immune receptors, including TLR3 and NF-κB pathway components [101]. This is accompanied by enrichment of N1 neutrophils within the TME, which exhibit increased expression of TLR3- and NF-κB-associated genes such as IL1RAP, ADAM8, TLR4, CX3CR1, FLOT1, and IL18 [101]. Together, these findings indicate that boiling histotripsy robustly engages innate inflammatory signaling networks, although context-dependent variability across tumor types remains an area of ongoing investigation.

Boiling histotripsy facilitates adaptive immune activation in part through preservation of non-denatured tumor antigens and enhanced antigen presentation by APCs within both the local TME and TDLNs [99]. This enables interaction with CD8^+^ and CD4^+^ T cells, supporting antigen-specific immune recognition and expansion locally and systemically (Table 1). In melanoma, boiling histotripsy increases tumor antigen presence across multiple immune populations within TDLNs, including B cells, macrophages, dendritic cells, monocytes, and granulocytes within 24 h post-treatment [99]. These findings support a model in which cell-free tumor debris traffics to lymphoid tissue, where dendritic cells mediate antigen uptake and presentation within the TDLN microenvironment [99]. In contrast, colorectal cancer models demonstrate increased dendritic cell accumulation in both the TME and TDLNs following boiling histotripsy, suggesting tumor- and parameter-dependent variability in antigen presentation pathways [99]. However, transcriptomic analyses in pancreatic cancer and renal cell carcinoma also show suppression of adaptive immune signaling pathways, alongside increased expression of anti-inflammatory cytokines such as IL-10 and IL-13 [101,102]. These contrasting findings suggest that although boiling histotripsy consistently promotes antigen release and immune cell recruitment, the downstream adaptive immune response may be shaped by intrinsic differences in tumor biology, baseline immune composition, and the post-ablation cytokine milieu. Consequently, while boiling histotripsy demonstrates considerable potential to enhance antitumor immunity, its capacity to induce a sustained “cold-to-hot” transition may not be universal across tumor types. Additional mechanistic studies are therefore needed to define the factors governing these divergent immune responses and to determine the generalizability of boiling histotripsy as an immunomodulatory strategy.

Boiling histotripsy has demonstrated the ability to influence systemic tumor control through induction of abscopal-like effects [99,102]. In melanoma models, treatment of primary tumors results in slowed growth of contralateral lesions and improved survival compared to controls, consistent with systemic immune activation [99]. These findings are particularly significant because they suggest that the immunological consequences of boiling histotripsy are not confined to the treatment site. Instead, local tumor disruption may generate systemic anti-tumor responses capable of recognizing and suppressing distant, untreated disease, highlighting the potential of boiling histotripsy to function as both an ablative and immune-modulating therapy (Table 1). Similarly, renal cell carcinoma models show increased CD8^+^ T-cell infiltration in both treated and distant tumors following boiling histotripsy, further supporting systemic immune involvement [102]. The presence of cytotoxic T cells within untreated lesions suggests that boiling histotripsy may promote the generation and trafficking of tumor-reactive effector cells, providing a potential mechanism for the control of metastatic disease beyond the primary treatment site.

Boiling histotripsy has also been evaluated in combination with immunotherapeutic strategies, including oncolytic viruses and immune checkpoint-modulating agents. In pancreatic cancer, boiling histotripsy combined with oncolytic reovirus enhances innate immune gene expression, increases neutrophil activation and NLR signaling, and promotes infiltration of NK cells and cytotoxic T-cell subsets while reducing regulatory T cells compared with monotherapy [101]. These changes suggest a shift toward a more immunostimulatory TME that may improve anti-tumor immune function and overcome barriers to effective immune-mediated tumor control. In melanoma, boiling histotripsy combined with CD40 agonism enhances antigen presentation, promotes CD8^+^ IFN-γ^+^ T-cell recruitment, and reduces immunosuppressive TGF-β2 [100]. These findings highlight the potential for boiling histotripsy-based combination strategies to amplify adaptive anti-tumor immunity by simultaneously strengthening immune activation and reducing mechanisms of immune suppression (Table 1). Triple combination therapy with checkpoint blockade further enhances systemic tumor control, including contralateral tumor attenuation consistent with an abscopal effect [100]. The current evidence indicates that boiling histotripsy-mediated immune priming may sensitize tumors to checkpoint inhibition, enabling local treatment to generate immune responses capable of targeting distant, untreated disease. Although boiling histotripsy consistently promotes immune activation across preclinical models, the magnitude and nature of these responses appear to be influenced by tumor context. Enhanced dendritic cell activation and antigen trafficking have been observed in melanoma models [99], whereas pancreatic and renal cell carcinoma models have demonstrated suppression of select adaptive immune signaling pathways despite robust innate inflammatory activation [101,102]. Collectively, these observations underscore the need for additional mechanistic studies to better define the tumor-specific factors governing immune responses following boiling histotripsy and to support broader clinical translation. Overall, boiling histotripsy integrates mechanical tumor destruction with preservation of antigenic integrity, enabling coordinated activation of innate and adaptive immune responses and supporting its continued investigation as a platform for both localized and systemic cancer therapy.

## 5. Shock Scattering Histotripsy

In contrast to intrinsic threshold histotripsy, cavitational tissue ablation can also be achieved through a shock-scattering mechanism when the peak negative pressure does not exceed the intrinsic cavitation threshold [12]. Shock-scattering histotripsy utilizes multi-cycle ultrasound pulses (typically 3–20 cycles) to stochastically nucleate and expand cavitation bubbles from preexisting nuclei within the initial cycles (Table 1). Once formed, sufficiently large bubbles function as pressure-release surfaces, enabling the subsequent positive-phase shock front, generated through nonlinear propagation, to impinge upon and scattering from the bubble interface [13]. This interaction produces constructive interference between incident and backscattered waves, yielding localized negative pressures that exceed the intrinsic cavitation threshold [12]. The resulting self-reinforcing bubble cloud is typically larger and denser than that produced by intrinsic threshold histotripsy, effectively increasing ablative capacity at the expense of spatial precision [14]. Rapid bubble expansion and collapse subsequently generate mechanical stresses that fractionate targeted tissue into acellular debris [15]. Within tumors, this process results in homogenization of treated regions, which may enhance immune accessibility by disrupting physical barriers to infiltration [103,104]. Although the immunologic consequences remain incompletely defined, the extent of mechanical disruption suggests substantial potential for immunomodulatory effects.

While most oncologic studies of shock-scattering histotripsy have focused on feasibility and ablation efficacy, emerging preclinical data indicate rapid and localized remodeling of tumor architecture following treatment. Across tumor models, shock-scattering exposure produces sharply demarcated regions of cavitation-induced fractionation, characterized by destruction of stromal structure and loss of organized tumor architecture. In an orthotopic ACE-1 canine prostate tumor model, transabdominal shock-scattering histotripsy induced central tumor necrosis with focal hemorrhage observed both immediately and at 3-week follow-up [104]. Comparable findings were reported in an orthotopic VX-2 rabbit renal tumor model, where treated regions demonstrated extensive acellular debris formation with clear demarcation from surrounding viable tissue [103]. These observations were further supported in follow-up analyses examining post-treatment tumor behavior and metastatic outcomes [105].

Beyond direct cytoreduction, these studies suggest that shock-scattering histotripsy alters the physical and antigenic landscape of the tumor microenvironment by disrupting stromal barriers and liberating intracellular and tumor-associated contents. This remodeling may increase the accessibility of tumor-derived antigens and thereby create conditions permissive for immune engagement (Table 1). However, the extent to which these released materials contribute to downstream immune activation remains an area of ongoing investigation. Bubble cloud formation and ablation efficiency are strongly influenced by treatment parameters, including pulse structure, cycle number, repetition frequency, and waveform nonlinearity [12], as well as by intrinsic tissue mechanical properties [106,107,108]. In stiffer or more fibrotic tumors, limited bubble expansion may reduce treatment efficacy. For example, ex vivo studies of human uterine fibroids demonstrated small, inconsistent cavitation zones with minimal histologic disruption and no clear apoptotic signatures [109]. Similarly, in the ACE-1 model, structural disruption was largely confined to focal hemorrhagic regions in the tumor periphery [104]. Collectively, these findings suggest that microenvironmental remodeling induced by shock-scattering histotripsy is not uniform across tumor types and is likely constrained by stromal density and mechanical heterogeneity.

The innate immune consequences of shock-scattering histotripsy may arise primarily from cavitation-induced mechanical injury and the subsequent release of DAMPs into the local TME (Table 1) [103]. Histological analyses in VX-2 renal and ACE-1 prostate tumor models support the development of an acute inflammatory response following treatment, including hemorrhage and immune cell infiltration consistent with injury-driven signaling [103,104]. Marked neutrophilic infiltration has been observed within fractionated tumor regions at 24 h post-treatment, whereas untreated tumors exhibit limited leukocyte penetration at tumor margins [103,105]. Given that shock-scattering histotripsy generates relatively large cavitation clouds, it remains unclear whether the magnitude, spatial distribution, or persistence of DAMP release differs from other histotripsy modalities (Table 1). Likewise, whether cavitation dynamics themselves modulate the intensity or composition of the innate inflammatory response is not yet defined. Overall, direct mechanistic characterization of innate immune signaling in shock-scattering cancer applications remains limited, with current understanding largely derived from histopathologic observations.

Adaptive immune consequences of shock-scattering histotripsy remain the least characterized aspect of cavitational ablation modalities. In chronic ACE-1 tumor studies, histological evaluation revealed multifocal lymphoplasmacytic inflammation three weeks following treatment [104], suggesting the potential for sustained immune infiltration beyond the acute injury phase. The presence of lymphoid and plasma cell populations is consistent with prolonged antigen exposure and raises the possibility that tumor-derived antigens released during treatment may remain immunologically accessible. However, definitive evidence linking these findings to antigen-specific adaptive immune activation remains lacking. Taken together, the limited number of mechanistic studies available suggests that shock-scattering histotripsy has the potential to promote both innate and adaptive immune responses; however, direct evidence defining these processes remains sparse. Much of the current understanding is inferred from histopathologic observations rather than comprehensive immunological analyses, highlighting the need for immune phenotyping and longitudinal functional studies to establish how cavitation-induced tissue disruption shapes durable antitumor immunity [103,104,105].

Although less immunologically characterized than the intrinsic threshold approach, cancer-related studies in shock-scattering histotripsy applications have influenced important considerations for the clinical translation of cavitational histotripsy for cancer treatment. Evidence from investigating the post-treatment metastatic burden of the VX-2 model suggested that histotripsy treatment did not significantly promote metastatic dissemination despite mechanical disruption, which was a key concern for the therapy given mechanical fragmentation potentially enhancing tumor spread [105]. As such, it is likely that this work influenced the histotripsy application for treating human renal-residing tumors in the #HOPE4KIDNEY clinical trial, recently applying for FDA approval as of May 2026. Shock-scattering histotripsy has also suggested how patient-specific tumor biomechanics may influence parameter optimization and treatment responsiveness given tissue stiffness limitations on bubble cloud expansion and treatment efficacy [106,107].

## 6. Low-Intensity Focused Ultrasound (LIFU)

Low-intensity focused ultrasound (LIFU) has emerged as a highly versatile, non-ablative focused ultrasound modality with expanding applications in both neuromodulation and oncology [16,17]. In contrast to the thermally driven tissue destruction associated with HIFU, LIFU utilizes lower-intensity, pulsed ultrasonic waves to non-invasively target either anatomical structures, including superficial and deep brain regions, or exogenously administered agents such as microbubbles and phase-transition nanodroplets (Table 1). Although no universally accepted intensity threshold defines LIFU, oncologic applications commonly employ acoustic exposures below approximately 3 W/cm^2^. Rather than producing extensive thermal injury, LIFU primarily induces transient mechanical and functional perturbations that evolve and resolve over hours to days following treatment [18]. For context, the U.S. Food and Drug Administration (FDA) defines non-significant risk (NSR) diagnostic ultrasound exposure limits as a spatial-peak temporal-average intensity (ISPTA) of ≤720 mW/cm^2^ for peripheral applications and ≤94 mW/cm^2^ for cephalic applications, with a spatial-peak pulse-average intensity (ISPPA) limit of ≤190 W/cm^2^ for all diagnostic ultrasound applications [110]. Accordingly, LIFU is more appropriately distinguished by its non-ablative biological effects than by a single intensity threshold.

Although the precise mechanisms underlying LIFU-mediated bioeffects remain incompletely defined and highly parameter-dependent, proposed mechanisms include reversible modulation of mechanosensitive ion channels, sonoporation-induced increases in membrane permeability, microtubule resonance, and microcavitation generated in combination with intravenously administered microbubbles. These effects are particularly important for transient disruption of the BBB and blood–tumor barrier (BTB), enabling enhanced penetration of therapeutics and immune modulators into otherwise poorly accessible tumors (Table 1). Consequently, LIFU-based approaches are increasingly being investigated as platforms for localized tumor microenvironment modulation, therapeutic sensitization, and coordinated innate and adaptive immune activation without extensive tissue destruction (Figure 1). Highly immunosuppressive and structurally restrictive TMEs, including anatomical barriers such as the BBB and BTB, can significantly limit immune infiltration and therapeutic penetration. As a result, many LIFU-based strategies have focused on transiently increasing vascular permeability and facilitating localized delivery of immune-modulating agents through combinations with microbubbles or acoustic droplet vaporization (ADV) systems. Although LIFU does not primarily rely on tissue ablation, its downstream effects on immune signaling and therapeutic responsiveness fit within the conceptual framework presented in Figure 1.

In a glioblastoma model, LIFU-guided sequential delivery of CXCL10 and IL-2 using an “open-source throttling” strategy enhanced intratumoral localization of CD8^+^ T cells while reducing T-cell exhaustion, effects that were further potentiated by anti-programmed cell death-ligand 1 (α-PD-L1) therapy [111]. Notably, LIFU alone did not produce detectable blood–brain barrier opening across the ultrasound intensities evaluated, whereas BBB disruption and subsequent therapeutic delivery required the combination of LIFU with CXCL10-coated microbubbles and IL-2/anti-PD-L1-loaded nanodroplets [111]. These findings suggest that the observed immunological effects are primarily attributable to the integrated ultrasound-assisted delivery platform rather than LIFU as a standalone intervention. Similarly, in melanoma, LIFU-triggered acoustic droplet vaporization (ADV) of a nanodroplet platform delivering the autophagy inhibitor SAR405 promoted development of a pro-inflammatory tumor microenvironment characterized by increased recruitment of natural killer cells and CD8^+^ T cells following upregulation of chemokines including CCL5 and CXCL10 (Table 1) [112]. Although an in vivo LIFU-only treatment arm was not included, in vitro experiments demonstrated that LIFU exposure alone did not significantly affect B16-F10 cell viability or apoptosis relative to untreated controls. Instead, LIFU primarily functioned as a stimulus for acoustic droplet vaporization, markedly accelerating SAR405 release from the nanodroplet platform compared with minimal passive drug leakage in the absence of ultrasound. Complementing these immune trafficking approaches, LIFU combined with microbubble-targeted destruction has also been shown to mechanically disrupt tumor-associated vasculature in a 4T1 murine mammary carcinoma model. This strategy reduced tumor blood perfusion while increasing dendritic cell and CD8^+^ T-cell populations within both tumors and tumor-draining lymph nodes [113]. Importantly, the inclusion of LIFU-only and microbubble-only control groups demonstrated minimal antitumor or immunological activity in the absence of combination treatment. Rather, the observed immune activation and tumor remodeling were associated with the synergistic interaction between LIFU and microbubble-mediated mechanical effects, highlighting the role of ultrasound as a facilitator of therapeutic bioactivity rather than an independent immunomodulatory intervention.

Activation of inflammatory signaling pathways, induction of ICD, and reprogramming of suppressive myeloid populations represent key components of the innate immune response generated by LIFU and its combination therapies. A recurring feature across these studies is the induction of acute cellular stress and subsequent release of DAMPs following LIFU-mediated mechanical disruption (Table 1) [114,115]. In a 4T1 mammary carcinoma model, LIFU-triggered phase-transformation nanoparticles induced multiple ICD-associated markers, including calreticulin surface exposure, HMGB1 release, and extracellular ATP secretion following treatment [114]. Notably, LIFU alone produced minimal antitumor or immunological activity, with outcomes comparable to vehicle-treated controls, whereas the combination strategy promoted ICD-associated signaling and immune activation. Similarly, in a KPC pancreatic cancer model, LIFU combined with microbubbles facilitated nuclear-to-cytoplasmic translocation of HMGB1 in tumor cells two days post-treatment, suggesting tumor cell damage [115]. These DAMPs are known to stimulate antigen-presenting cells, promote dendritic cell maturation, and activate downstream inflammatory signaling pathways through pattern recognition receptor engagement. Likewise, iRGD-modified phase-transition liposomes loaded with slow-release STING agonists enhanced type I interferon signaling alongside dendritic cell maturation and activation within the same model (Table 1) [116]. Although LIFU exposure facilitated acoustic droplet vaporization and localized payload release, the enhanced innate immune responses were observed in conjunction with the STING-loaded liposome platform rather than ultrasound treatment alone.

Beyond local inflammatory remodeling and innate immune activation, LIFU-based therapeutic strategies have also been shown to enhance adaptive anti-tumor immunity, particularly through modulation of CD8^+^ T-cell responses and reversal of immune escape mechanisms. Several studies have specifically investigated the ability of LIFU combinations to improve cytotoxic T-cell recruitment, activation, and responsiveness to immune checkpoint blockade (Table 1). In the GL261 glioma model, combination treatment using LIFU with fluorescein-mediated sonodynamic therapy induced robust CD8^+^ T-cell infiltration alongside significant depletion of myeloid-derived suppressor cells, ultimately resulting in improved survival compared to fluorescein or focused ultrasound treatment alone [117]. Similarly, LIFU-induced nanodroplet vaporization promoted autophagic stress and pro-inflammatory signaling that enhanced CD8^+^ T-cell infiltration and sensitized tumors to α-PD1 therapy [112]. Mechanistically, these effects are thought to arise from increased antigen release and amplification of inflammatory signaling following LIFU-mediated phase transition events, thereby improving immune checkpoint blockade efficacy. These findings suggest that LIFU-based approaches can reshape immunosuppressive tumor microenvironments and restore cytotoxic T-cell activity, particularly when integrated with immunogenic stressors and checkpoint inhibition strategies (Table 1). The expanding application of LIFU in combination with drug delivery systems and immunotherapies has demonstrated considerable promise for remodeling the tumor microenvironment. However, because many studies evaluate LIFU alongside adjunctive therapeutic platforms rather than as a standalone intervention, the specific contribution of ultrasound-mediated bioeffects to the observed immune responses remains difficult to define [111,112,114,115,116,117,118]. Further mechanistic studies will be important to optimize these combination strategies and facilitate broader clinical translation.

Clinical translation of oncologic LIFU applications has accelerated in recent years, particularly in neuro-oncology, where its ability to transiently disrupt the BBB has enabled delivery of therapeutics that would otherwise exhibit poor CNS penetration. Ongoing clinical studies are evaluating MRI-guided LIFU in combination with sonodynamic therapy agents such as SONALA-001 and orally administered 5-aminolevulinic acid (5-ALA) for recurrent or progressive glioblastoma, with the goal of selectively activating tumor sensitizers while minimizing off-target tissue injury [119]. In parallel, LIFU-mediated BBB opening is being investigated to enhance immune cell trafficking for adoptive cellular immunotherapies, including anti-EGFR bispecific T-cell approaches in glioblastoma [120]. A related preclinical study employing low-intensity pulsed ultrasound (LIPU), rather than the focused LIFU platforms discussed above, demonstrated that BBB disruption significantly increased CAR T-cell delivery into the CNS and enhanced survival compared with CAR T-cell therapy alone [121]. Similarly, combining LIPU-mediated BBB opening with anti-PD-1 therapy or CXCL10-secreting antigen-presenting cells improved T-cell infiltration into gliomas, prolonged survival, and generated durable antitumor immunity following tumor rechallenge, highlighting the potential for transient BBB opening to facilitate both immune cell trafficking and immunotherapeutic efficacy within the CNS [121]. Additional trials are exploring LIFU-mediated BBB disruption to improve delivery of standard-of-care therapies for brain metastases arising from non-small cell lung cancer [113]. These trials highlight the expanding translational role of LIFU as a non-ablative platform for enhancing therapeutic delivery, improving immune infiltration, and sensitizing tumors to emerging immunotherapeutic strategies. More broadly, they demonstrate how LIFU can be leveraged to overcome anatomical and physiological barriers that have historically limited effective treatment of central nervous system malignancies.

## 7. Conclusions

Focused ultrasound encompasses a broad and rapidly evolving spectrum of therapeutic technologies that differ in their physical mechanisms but share a common ability to non-invasively target tissue with high spatial precision. Across thermal, mechanical, and low-intensity approaches, these modalities have progressed from experimental tools for local tumor control to clinically relevant interventions with expanding biological and therapeutic implications. A central theme emerging from the literature is that focused ultrasound is no longer defined solely by its ability to ablate tissue. Instead, it is increasingly recognized for its capacity to influence the tumor microenvironment and engage the immune system in ways that may extend its impact beyond the treated lesion. This shift in perspective has positioned focused ultrasound as a versatile platform that bridges local intervention with systemic biological effects.

## 8. Future Directions

While focused ultrasound modalities have demonstrated considerable potential for both tumor ablation and immune modulation, important knowledge gaps remain regarding the mechanisms that drive these responses. Future studies should focus on directly comparing modalities under standardized conditions to better understand how differences in thermal and mechanical bioeffects influence immune activation, antigen presentation, and systemic anti-tumor responses. Additionally, optimization of treatment parameters will be critical for maximizing therapeutic efficacy and reproducibility across tumor types. Advances in technologies such as single-cell sequencing, spatial transcriptomics, and multiplex imaging provide new opportunities to characterize focused ultrasound-induced immune responses at greater resolution. Coupled with ongoing clinical translation, these approaches may help identify biomarkers of response, guide patient selection, and facilitate the integration of focused ultrasound into multimodal cancer treatment strategies. Ultimately, a deeper understanding of modality-specific biological effects will be essential for fully realizing the therapeutic potential of focused ultrasound in oncology and beyond.

## Figures and Tables

**Table 1:** Summary of reported immune responses associated with focused ultrasound modalities. Comparison of experimentally reported effects of high-intensity focused ultrasound (HIFU), intrinsic threshold histotripsy, boiling histotripsy, shock-scattering histotripsy, and low-intensity focused ultrasound (LIFU) on the tumor microenvironment, innate immune activation, adaptive immune responses, systemic anti-tumor immunity, and immunotherapy synergy. Entries summarize experimentally reported immune effects described in the cited literature for each focused ultrasound modality.

Immune Feature	HIFU	Intrinsic Threshold Histotripsy	Boiling Histotripsy	Shock-Scattering Histotripsy	LIFU
Primary Mechanism	Thermal + mechanical injury	Cavitation-mediated mechanical fractionation	Boiling bubble-mediated mechanical emulsification	Shock-scattering cavitation fractionation	Non-ablative mechanical modulation
Antigen Preservation	Moderate	High	High	Not reported	Not reported
DAMP Release	✓	✓	✓	Not reported	✓
Innate Immune Activation	PRR, inflammasome, cytokine signaling	Strong DAMP-PRR signaling	Strong inflammatory cytokine response	Acute inflammatory cell infiltration	ICD and STING-associated signaling
TME Remodeling	Cold → Hot	Cold → Hot	Cold → Hot	Structural disruption	Enhanced permeability and immune trafficking
Adaptive Immune Activation	CD4^+^/CD8^+^ T-cell responses	Robust CD8^+^ T-cell responses and memory	T-cell priming and expansion	Lymphoplasmacytic infiltration reported	Enhanced CD8^+^ T-cell infiltration
Reduction in Immunosuppression	↓ Tregs, IL-10, VEGF-A	Reported	M2 macrophages ↓, Tregs ↓	Not reported	MDSCs ↓
Systemic Immune Effects	Antigen-specific immunity	Abscopal-like effects and memory	Abscopal-like effects	Not reported	Primarily in combination therapies
Immunotherapy Synergy	Checkpoint inhibitors	Checkpoint inhibitors	Checkpoint inhibitors, CD40 agonists, oncolytic viruses	Not reported	Checkpoint inhibitors, STING agonists

Abbreviations: DAMP, damage-associated molecular pattern; ICD, immunogenic cell death; LIFU, low-intensity focused ultrasound; PRR, pattern recognition receptor; TME, tumor microenvironment; Treg, regulatory T cell; MDSC, myeloid-derived suppressor cell.

## Data Availability

No new data were created or analyzed in this study. Data sharing is not applicable to this article.

## Abbreviations

ADV	Acoustic droplet vaporization
AIM2	Absent in melanoma 2
APCs	Antigen-presenting cells
ATP	Adenosine triphosphate
BBB	Blood–brain barrier
BTB	Blood–tumor barrier
CCL5	C-C motif chemokine ligand 5
CD	Cluster of differentiation
CNS	Central nervous system
CRT	Calreticulin
CXCL10	C-X-C motif chemokine ligand 10
DAMPs	Damage-associated molecular patterns
DCs	Dendritic cells
EGFR	Epidermal growth factor receptor
FasL	Fas ligand
FUS	Focused ultrasound
GzB	Granzyme B
HCC	Hepatocellular carcinoma
HIFU	High-intensity focused ultrasound
HMGB1	High mobility group box 1 protein
HSPs	Heat shock proteins
ICD	Immunogenic cell death
IFN-γ	Interferon-gamma
IL	Interleukin
LIFU	Low-intensity focused ultrasound
MAPK	Mitogen-activated protein kinase
MHC II	Major histocompatibility complex class II
MRI	Magnetic resonance imaging
NDL	Neu exon deletion line
NF-κB	Nuclear factor kappa B
NK cells	Natural killer cells
NLRs	NOD-like receptors
NLRP3	NLR family pyrin domain containing 3
PD-1	Programmed cell death protein 1
PD-L1	Programmed cell death-ligand 1
Pf	Perforin
PRRs	Pattern recognition receptors
PSA	Prostate-specific antigen
PSMA	Prostate-specific membrane antigen
RIG-I-like receptors (RLRs)	Retinoic acid-inducible gene I-like receptors
SAR405	Selective autophagy inhibitor 405
STING	Stimulator of interferon genes
TAAs	Tumor-associated antigens
TDLNs	Tumor-draining lymph nodes
TGF-β2	Transforming growth factor beta 2
Th1	T helper type 1
TLRs	Toll-like receptors
TME	Tumor microenvironment
TNF	Tumor necrosis factor
TNF-α	Tumor necrosis factor-alpha
Tregs	Regulatory T cells
VX-2	Shope virus-induced rabbit carcinoma model
α-PD-1	Anti-programmed cell death protein 1
α-PD-L1	Anti-programmed cell death-ligand 1
α-CTLA-4	Anti-cytotoxic T-lymphocyte-associated protein 4
